# Strength-based technology clubs for autistic adolescents: A feasibility study

**DOI:** 10.1371/journal.pone.0278104

**Published:** 2023-02-03

**Authors:** Matthew Jones, Benjamin Milbourn, Marita Falkmer, Tele Tan, Sven Bölte, Sonya Girdler

**Affiliations:** 1 Curtin School of Allied Health, Faculty of Health Sciences, Curtin University, Perth, WA, Australia; 2 Curtin Autism Research Group (CARG), Curtin University, Perth, WA, Australia; 3 Swedish Institute for Disability Research, School of Education and Communication, Jönköping University, Gjuterigatan, Sweden; 4 School of Electrical Engineering, Computing and Mathematical Sciences, Faculty of Science and Engineering, Curtin University, Perth, WA, Australia; 5 Center of Neurodevelopmental Disorders (KIND), Centre for Psychiatry Research, Division of Neuropsychiatry, Department of Women’s and Children’s Health, Karolinska Institutet & Child and Adolescent Psychiatry, Stockholm Health Care Services, Stockholm County Council, Stockholm, Sweden; 6 School of Allied Health, University of Western Australia, Perth, Western Australia, Australia; The University of Sydney, AUSTRALIA

## Abstract

Strength-based technology clubs are thought to help autistic adolescents transition to adulthood by developing positive traits, enhancing technical skills, and creating supportive networks. A newly developed strength-based technology club was delivered to 25 autistic adolescents, with the feasibility tested via qualitative and quantitative methods. Autistic adolescents, their parents, and club facilitators participated in separate focus groups, with audio data transcribed and thematically analyzed. Quantitative data was collected via adolescent and parent-reported pretest-posttest measures following the 15-week program. Autistic adolescents were highly satisfied with the club *(acceptability)*, the technology club satisfied an unmet need (*demand*), with the program demonstrating the potential to be integrated into the current therapy system in Australia (*integration*). Feasibility areas that could be improved in delivering future clubs are discussed.

## Introduction

To date, interventions aimed at improving outcomes for autistic individuals have primarily targeted their social communication and behavior challenges [1–3], and despite significant investment, outcomes in adulthood often remain poor [4]. Internationally, autistic individuals experience low and underemployment [5–7], a high prevalence of anxiety [8], more loneliness, and have poorer friendship quality than their neurotypical peers [9–12]. Notably, a study involving 235 autistic adolescents (*n* = 185) and adults (*n* = 50) found that 46.4% had no ‘same-aged’ friends with whom they participated in social or recreational activities [13]. Low levels of engagement in activities outside of school and home have been noted as contributing to poor outcomes for school-aged autistic youth [14, 15]. It has been proposed that increasing autistic youth participation in activities outside of school and home may be vital in fostering better outcomes into adulthood [15].

Participating in activities outside of school and home is crucial in promoting adolescents’ social, emotional, and physical development [15–17]. Out-of-school activities vary widely and include sport, leisure, volunteering, extracurricular, or youth programs [18]. For adolescents without a disability, out-of-school activities are associated with developing social relationships with similarly aged peers [19–21] and non-familiar adults [19], improved social skills [20, 22], forming a personal identity [19, 23, 24], low risk of depressive symptoms [17, 24, 25], and reduced loneliness [25]. Out-of-school activities appear to have similar benefits for adolescents with a disability, being associated with increased levels of self-determination [26], a sense of belonging, and wider social networks [26, 27]. For autistic adolescents, involvement in extracurricular activities is associated with fewer depressive symptoms [28], making friends, belonging to a community, and the opportunity to practice social skills [29].

While the weight of evidence makes it clear that out-of-school activities are beneficial to all adolescents, regardless of their disability status, autistic adolescents participate in few out-of-school activities [14]. Autistic adolescents are more likely than others to engage in solitary activities rather than join in with broader community activities [15, 30]. A survey of 11280 adolescents (13–16 years old) with disabilities found that of all disability groups represented in the survey, autistic youth most frequently reported never seeing friends outside of school (43.3%) and not participating in non-school activities (43.8%) [14]. This pattern of limited participation in community activities for autistic individuals continues into adulthood [31, 32].

While participating in out-of-school activities is associated with several benefits for autistic adolescents, participation does not guarantee positive outcomes. For youth without disability, benefiting from out-of-school activities depends on the program engaging them psychologically and capturing their full attention [16]. Parents of autistic adolescents report that technology-based activities are particularly engaging and motivating for their children [33], with previous technology clubs including 3D design programs [33, 34], game development, graphic design, and animation and photography [35] found to be enjoyable and motivating. Given these findings, it seems reasonable to suggest that autistic adolescents would find a technology-based out-of-school program engaging and enjoyable.

Technology-based activities extend beyond providing an engaging medium and potentially align with the recognized strengths of autistic individuals. Previous studies selected visual-based tasks, such as 3D design activities, to leverage the visual perceptual strengths seen in some autistic individuals [33–35]. Other noted strengths of autistic individuals, such as attention to detail, are also advantageous for employment in the Information and Communication Technology (ICT) sector [36]. The ICT sector has attempted to leverage the strengths of autistic individuals through specialized employment programs that match strengths to specific technology-based tasks [37–39]. Given the potential alignment between technology and recognized strengths, technology clubs have emerged in the field of autism as a strength-based approach to explore ICT career pathways, promote social engagement and build technical skills for employment [35, 40]. Technology-based activities may provide an ideal out-of-school activity that contributes to positive psychological development and more directly to improving vocational outcomes for autistic adolescents.

While strength-based technology clubs appear to be a logical approach to engaging autistic adolescents in out-of-school activities, many models of service could be adopted in delivering such programs. To date, technology programs describing themselves as strength-based have incorporated special interests into activities [35], leveraged strengths through visually-based tasks [33], prioritized skill development over remediating deficits [34], and applied positive psychology principles [40]. With the goal of developing a standardized evidence-based framework for underpinning the delivery of strength-based technology programs to autistic adolescents, we undertook a line of research guided by the Medical Research Council (MRC) framework for developing complex interventions [41]. The MRC framework outlines the stages of developing a complex health intervention as: 1) theory development; 2) feasibility; 3) evaluation; and 4) implementation [41]. Theory development focused on identifying change processes by identifying the active ingredients of strength-based technology clubs for autistic adolescents. In fulfilling this requirement, a previously conducted systematic review of the literature combined with a qualitative investigation of three established strength-based technology clubs [42] informed the development of the IVAR (interests, value, autonomy, and requirements)strength-based framework. The IVAR strength-based framework provides recommendations and strategies for designing and delivering strength-based technology clubs within four areas: interests, value, autonomy, and requirements (IVAR). The aim of this study focused on feasibility testing and preliminary evaluation of a measurement framework to understand the utility of strength-based technology club for autistic adolescents.

## Method

### Design

Informed by the feasibility testing framework from Bowen et al. [43], the present study considered the feasibility areas of *acceptability*, *demand*, *implementation*, *practicality*, *adaptation*, *integration*, *expansion*, and *preliminary efficacy* testing. Bowen’s framework [43] provides as a series of questions to inform feasibility testing. Qualitative and quantitative methods were used to investigate and evaluate each aspect of feasibility (Table 1).

**Table 1:** Methods to investigate feasibility based on Bowen et al. [43].

Area of focus	Definition	Methodology
Acceptability	Participant’s satisfaction with the intervention	Qualitative approach with data collected via focus groups and interviews
Demand	The demand for intervention within the autistic population	Qualitative approach with data obtained via focus groups and interviews
		Recruitment and retention rates
Implementation	How the intervention is delivered	See ‘Program’ section for details
Practicality	Ability to deliver the intervention with available resources	Qualitative approach with data obtained via focus groups and interviews (Facilitator data only)
Adaptation	Modifications made to the intervention	Qualitative approach with data collected via focus groups and interviews
Integration	Changes required to integrate the intervention into existing health and community infrastructure	Qualitative approach with data collected via focus groups and interviews
Expansion	Consideration as to how the intervention could be increased in scale or applied to a different population/setting	Qualitative approach with data collected via focus groups and interviews
Preliminary efficacy	Limited efficacy testing and evaluation of measurement framework	Quantitative evaluation through adolescent and parent-reported measures
		Qualitative approach with data obtained via focus groups and interviews (perceived outcomes)

### Program

A strength-based technology club for autistic adolescents was created and delivered using the IVAR framework’s four areas: interests, value, autonomy, and requirements. Interests related to strategies that leveraged the pre-existing interests of autistic adolescents. For example, when teaching video game design, facilitators would incorporate autistic adolescents’ interests into designing the video game. Value related to building competence and skills unique to the individual. For example, facilitators set individual technology goals with autistic adolescents rather than goals based on autism deficits. Autonomy is related to creating opportunities for autonomous behavior. For example, autistic adolescents were provided with a choice of technology-based activities. Requirements related to strategies that created compatible physical and social environments. For example, the technology-club was exclusively available for autistic adolescents, which created a supportive social environment.

A secondary school outside of Perth (Western Australia) Metropolitan area was selected as an appropriate venue for delivering the current program, with access provided to two teaching rooms and capacity for up to 30 students. The hardware, including laptops, mice, and programmable robots, provided by the research team, was secured with funding from a community grant. In line with the previously established recommendations for delivering technology clubs for autistic youth, volunteer facilitators with an interest and experience in technology-based activities were recruited from local volunteer community organizations and universities via word of mouth for the 14-week program. Before commencing the technology club, all facilitators participated in a four-hour training session covering topics aligned with our evidence-based recommendations, including environmental considerations, collaborative teaching approaches, strategies for incorporating choice and interests in activities and showcasing adolescent’s skills.

The technology club initially began with a holiday program held over four days, six hours per day, across one week. In contrast to the 14-week program, the holiday program was delivered by paid facilitators provided by an independent technology educator provider specializing in technology and coding for children in the community. This team were not familiar with disability and facilitators participated in the same training as outlined above and modified their standard curriculum to align with our recommendations for delivering strength-based technology clubs to autistic adolescents. During the holiday program, adolescents were supported in developing a video game using the software programs, Construct 2 [44] and Twine [45]. Construct 2 [44] is a free game-making program underpinned by a visually-based coding system that employs more images than words. Twine [45] is an open-source interactive storytelling tool supporting the development of a ‘choose your own adventure’ style story that can be shared with other players and played as a ‘text-adventure’ game, exploring multiple story plots.

Following the holiday program (four days over one week), the participants attended a two-hour technology class every Saturday for 14 weeks. Participants freely choose between four technology-based activities: Construct 2 [44], Twine [45], online computer coding activities, or robotics. A coding manual for Construct 2 with a focus on visual instructions was created to support student learning. Robotics involved building robots known as mBots [46] and controlling the robots through computer coding activities. At the beginning of each session, volunteer facilitators demonstrated a new technique that autistic adolescents could apply during the activity. For example, at the beginning of one session, participants were shown how to animate their video game characters and encouraged to incorporate this new technique into their game. The two-hour session was divided by a 15-minute snack break, during which participants were encouraged to socialize. The 14-week program was facilitated by the first author, with assistance from volunteer facilitators, including parents and community volunteers experienced or interested in technology-based activities.

### Participants

Adolescents were invited to participate in the research if they met the following criteria: a) aged between 10 and 18 years old; b) had a parent-reported autism spectrum disorder (ASD) diagnosis according to DSM-5 [47] or DSM-IV [48]; c) had an interest in computers and technology; and d) were able to read and converse in English. Adolescents could choose to attend the technology club without participating in the research project. Adolescents were excluded from the research program if they had a parent-reported intellectual disability diagnosis. Parents were invited to participate in the research if they: a) had a child whom they reported had an ASD diagnosis according to DSM-5 [47] or DSM-IV [48]; and b) were able to read and converse in English. Facilitators were invited to participate in research if they: a) were aged 18 years and older; b) had facilitated multiple technology club sessions; and c) were able to read and converse in English.

**Fig 1:**
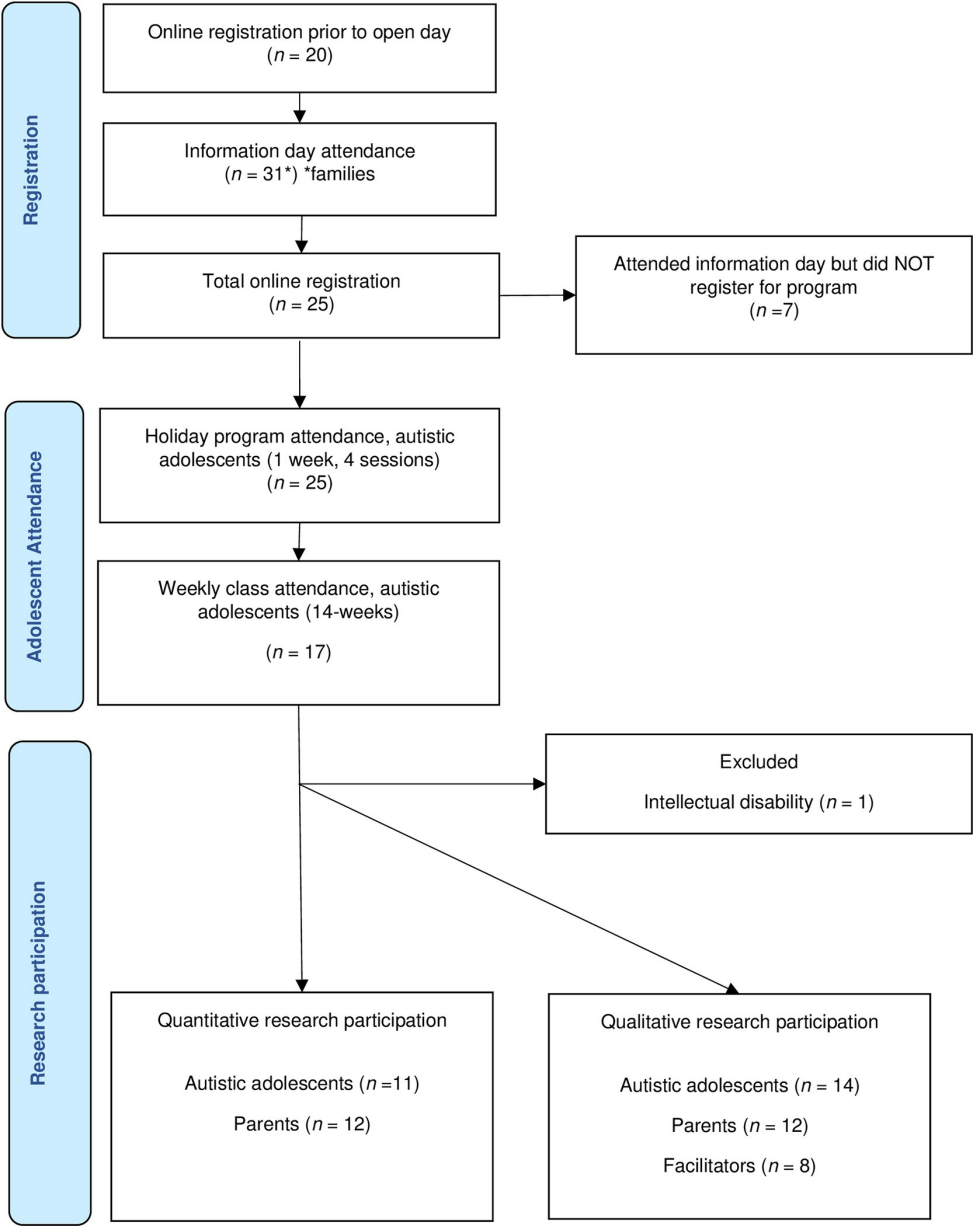
Participant participation and research recruitment process.

The technology club was advertised via a flyer that was distributed to the community via local schools, service providers, and social media, resulting in 20 registered autistic adolescents. An information session was subsequently held at a central building within the town; 31 families attended and received information on club activities, facilitators’ role, the research project, and parent testimonies from a previously investigated computer coding club [42]. Following the information day, the total online registrations increased to 25. Not all adolescents who participated in the technology club participated in the research project. Of the 25 registered adolescents, one was excluded from the research based on parent-reported intellectual disability, eight adolescents did not continue with the technology club following the holiday program, and five adolescents declined participation in quantitative data collection (Fig 1). In total, 11 adolescents completed all quantitative measures (Fig 1). One parent completed the parent-reported measures when their child did not, bringing quantitative parent participation to 12 (Fig 1). All five adolescents who did not complete the quantitative measures participated in the focus groups. Two adolescents who completed quantitative measures did not participate in the focus groups, bringing adolescent participation in focus groups to 14 (Fig 1). Due to time commitments, not all parents could participate in the focus groups bringing the total to 12. All eight facilitators participated in the focus groups.

The socio-demographics of adolescents, parents, and facilitators depending on participation are detailed in Tables 2–4. Adolescents had a mean age of 12.2 and 11.9 years for qualitative and quantitative data, respectively (Table 2). The majority of adolescent participants were male in qualitative and quantitative data (82% and 79%, respectively), with most adolescents demonstrating moderate autistic behaviors according to the SRS-2 (Table 2). Anxiety and ADHD were the equal highest comorbidity in both data sets (Table 2). Parents who participated in the research had a mean age of 44.7 and 43. 9 years for quantitative and qualitative data, respectively (Table 3). For quantitative data, most parents had completed a tertiary degree (42%), while for qualitative data, the highest level of completed education was a Technical and Further Education (TAFE) certificate (42%) (Table 3). The majority of parents were female for quantitative (83%) and qualitative data (100%). All facilitators participated in the focus group (*n* = 8), had a mean age of 41, with two facilitators (25%) reporting a diagnosis of autism, half with autistic children who attended the club, and most had an ICT or education background (37% each) (Table 4).

**Table 2:** Characteristics of adolescents who participated in technology clubs as reported by parents.

Characteristics	Pre-posttest participants	Focus group participants
Adolescents (n)	11	14
Age, mean (SD)(years)	12.2 (1.40)	11.9 (1.94)
Range, years	10–15	10–17
Diagnosis, n (%)		
Autism	10 (91%)	13 (93%)
PDD-NOS	1 (9%)	1 (7%)
SRS-2 (T-score)		
Within normal limits (30 - 59T)	1 (9%)	1 (7%)
Mild (60T - 65T)	3 (27%)	2 (14%)
Moderate (66T - 75T)	4 (36%)	5 (36%)
Severe (76T or higher)	3 (27%)	4 (29%)
Not reported		2 (14%)
Sex, n (%)		
Male	9 (82%)	11 (79%)
Female	2 (18%)	3 (21%)
Other medical conditions reported, n (%)		
NA	6 (55%)	9 (64%)
Anxiety	4 (36%)	4 (29%)
ADHD	4 (36%)	4 (29%)
Other	4 (36%)	4 (29%)
Schooling, n (%)		
Public	7 (64%)	7 (50%)
Private	3 (27%)	5 (36%)
Public with special needs	1 (9%)	2 (14%)

NA: Not Applicable; PDD-NOS: pervasive developmental disorders not otherwise specified; SRS-2: Social Responsiveness Scale, 2^nd^ Edition; ADHD: attention deficit hyperactivity disorder; Public school: Government funded education where tuition fees are covered by the relevant state or territory. Private school: non-government schools where students pay the tuition and other fees. Special needs support: A range of educational supports to allow a student with a disability to participate

**Table 3:** Characteristics of parents of adolescents who participated in technology clubs.

Characteristics	Quantitative participants	Qualitative participants
Parents (n)	12	12
Age, mean (SD)(years)	44.7 (7.40)	43.9 (6.43)
Range, years	33–57	33–57
Highest education, n (%)		
Secondary school	3 (25%)	3 (25%)
TAFE certificate	3 (25%)	5 (42%)
Tertiary degree (complete)	5 (42%)	3 (25%)
Tertiary degree (incomplete)	1 (8%)	1 (8%)
Sex, n (%)		
Male	2 (17%)	0
Female	10 (83%)	12 (100%)
Income (yearly before tax AUD) n (%)		
$18 000 - $40 000	2 (17%)	1 (8%)
$40 000 - $80 000	2 (17%)	2 (17%)
$80 000 - $180 000	5 (42%)	7 (58%)
$180 000 +	1 (8%)	0
Not disclosed	2 (17%)	2 (17%)

TAFE: Technical and Further Education; AUD: Australian Dollar

**Table 4:** Characteristics of facilitators who delivered computer coding classes.

Characteristics	Focus group participants
Facilitators (n)	8
Age, mean (SD)(years)	41 (8.12)
Range, years	25–53
Diagnosed with autism	
YES	2 (25%)
NO	6 (75%)
Child with autism, n (%)	
YES	4 (50%)
NO	4 (50%)
Child attends technology club (%)	
YES	4 (50%)
NO	4 (50%)
Previous experience working with autism	
No experience	5 (63%)
1–5 years	1 (12%)
5+ years	2 (25%)
Professional background, n (%)	
Engineering	1 (13%)
ICT	3 (37%)
Education	3 (37%)
Research	1 (13%)

ICT: Information and Communication Technology

Ethical approval was obtained from the Human Research Ethics Office (HRE2017-0147) at Curtin University, Perth, Western Australia. To avoid coercion, participation in the research was voluntary, and adolescents could attend the technology club without agreeing to participate in the research. Information sheets were provided, and informed written consent and verbal consent from parents, facilitators, and assent from adolescents was gained for those choosing to participate.

### Qualitative data collection

Data collection took place between October and December 2019 and evaluated a technology program delivered for 15-weeks (one-week intensive holiday program and 14 weeks of Saturday classes) in a city in Western Australia. Following completion of the program participants (autistic adolescents, their parents, and facilitators) took part in separate focus groups, with those unable to attend engaging in an individual interview. Focus groups were run on Saturdays between 10am and 2pm. Three focus groups were run with adolescents consisting of autistic adolescents who attended the club (focus group 1: 5 adolescents; focus group 2: 4 adolescents; focus group 3: 5 adolescents). Focus group 4 was run with 10 coding club facilitators. Focus group 5 comprised of 8 parents with a further 2 parents opting to participate in an interview. Questions were emailed to all participants up to a week prior to the group. Focus groups followed a discussion guide tailored to each participant group that aimed to explore the feasibility areas structured around the Bowen framework [43]. Areas of the framework included acceptability and demand, practicality and adaptation, implementation and adaptation, perceived outcomes and integration and expansion. Examples of the sort of questions included were: What did you enjoy? What would you like more of? What could be done more to support participation? What did you learn? (**See S1 File**).

To maximize adolescents’ engagement and understanding of the discussions and interviews, the adolescent discussion guide was supported by visual aids (**see S2 File**), adhering to published recommendations for conducting qualitative research with autistic individuals [49].

### Quantitative procedures

Sociodemographic data were collected from adolescents, parents, and facilitators. Adolescent’s autistic traits were assessed at pre-test via parent proxy reporting on the Social Responsiveness Scale–Second Edition (SRS-2) [50], a measure comprising of 65 items, providing an indicator of the severity of social deficits. The SRS-2 has strong psychometric properties [50] and good sensitivity [51]. Adolescents and parents completed pre-test measures prior to attending the technology club, with post-test measures completed in the following 15 weeks. Given the program was underpinned by the IVAR strength-based framework, which focused on developing competence, building autonomy, and creating supportive environments, the measurement framework was comprised of tools focused on measuring positive traits in preference to deficits. A focus was placed on self-efficacy and self-determination due to their predictive nature of positive outcomes in adulthood [52–54]. In autistic individuals, higher self-determination levels are associated with post-school education, employment, quality of life, and independent living [54, 55]. Friendship and loneliness scales were also included due to the previous studies documenting the social benefits of technology clubs for autistic adolescents [34, 35]. Due to the alignment of some autistic individuals’ strengths and STEM career pathways [55], a STEM career interest checklist was included. Finally, a quality-of-life assessment was included to investigate the technology club’s wider positive benefits (**see S3 File**).

### Data analysis

Standards for reporting qualitative data [56] was utilsed to inform qualitative data analysis. Audio recordings which were de-identified, pseudonyms allocated, transcribed and imported into Nvivo 12 [57]. The researchers adopted a constructionist paradigm to comprehend the sociocultural contexts informing the lived experience [58]. The use of this paradigm justified the need for adopting a coding framework based on Bowen’s et al. [43] areas of feasibility. Members of the research team were also guided by Braun and Clark’s [59] thematic process including generating themes within the eight areas of focus addressed by feasibility studies [43]. Assumptions of the researchers included the importance of person-centred practice [60] and the power of the social environment as a catalyst for change [61]. Each sentence from the interviews was read with consideration to the possible meanings considered in relation to the Bowen framework. Relevant statements were highlighted, selected, and coded. Newly coded data were then grouped into broad themes and further analysed in relation to Bowen’s framework [43].

Coding and themes were discussed by the research group weekly over a month period to check on relevance and appropriate thematic placing until consensus was reached. The results were summarized and then distributed back to participants for member checking, with no themes modified following the member checking results. See Table 5 for thematic map of codes mapped to Bowen framework [43].

**Table 5:** Thematic map of qualitative data.

Area of focus Bowen Framework [43]	Themes	• Sub themes with number of codes
Acceptability	• Safe environment • Types of activities	Activities (73) Friendship (13) Mentors (9) Physical environment (3) Safe environment (41)
Demand	Resources	Another holiday program (1) Duration (6) Fee (3) Limited previous opportunity (8) Longer duration (1) More hardware and software (13) More mentors (5) No other activities (2) Replace other services (1)
Practicality	• technology management • facilitator technical knowledge	All ASD (4) Autism specific training (1) Coding manual (2) Common activities (1) Flexibility to change activities (1) Interests (1) Loud room and quiet room (1) Overall goal (1) Role model (1) Step by step instructions (2) Transition periods (1)
Adaptation	• Understanding the individual • visual resources	Activities (24) Community integration (20) Environment (2) Logistics (10) Technical (3) Training (6)
Integration	• Fees • National Disability Insurance Scheme	
Expansion	neurotypical peers	
Outcomes	• making friends • socialize • develop positive traits	Felling proud (3) Increase confidence (10) Social skills engagement through activity (9)

Quantitative data from pre-test post-test measures were analyzed using the Statistical Package for Social Sciences (SPSS) Version 26.0 [62]. A Wilcoxon Signed-Rank test was performed to compare pre-test and post-test scores, with z scores provided. The rationale for using this test is the Wilcoxon signed-rank test is it is a more powerful than the sign test because it makes use of the magnitudes of the differences rather than just their signs [63].

## Results

### Qualitative findings

Focus groups with autistic adolescents, parents, and facilitators were employed in exploring all aspects of feasibility. Thematic analysis produced a total of 13 themes with two themes for *acceptability* (safe environment, type of activities) one theme for *demand* (resources), two themes for *practicality* (technology management and facilitator technical knowledge), two themes for *adaptation* (understanding the individual and visual resources), two themes for *integration* (Fees and National Disability Insurance Scheme), one theme for *expansion* (including neurotypical peers) and three themes for *outcomes* (making friends, socialize, develop positive traits) (Table 5).

#### Acceptability

Two themes supported participant satisfaction with the club: safe environment and type of activities. The theme of safe environment contributed to satisfaction with the program, with adolescents feeling there was no prospect of bullying, that they could be themselves, and that they belonged. While adolescents shared bullying stories from school, they reported bullying did not occur at the technology club because they *“all have a common interest in mind*.*”* Parents noted that adolescents at the club were not bullied for their “*quirks*” but valued for their “*differences*.” Facilitators felt that the club provided a safe environment because adolescents felt like they were “*with their people*” and in a place where they could “*be absolutely themselves*.” Finally, parents reported that belonging resulted from everyone sharing a diagnosis of autism, with one parent (who was also a facilitator) sharing the moment her child realized everyone at the technology club had autism.

*The kid was*, *‘Oh*, *hang on a minute*, *I’m not the only person in this classroom with autism*.*’ And that was*… *about week 2 or 3*, *that Rachel actually clicked that every single other child was on the spectrum*, *and she wasn’t just this lone figure in the classroom that’s got this label on her*. *So that was an interesting shift and I think that was sort of around the time when she went*, *‘Oh my god*, *these are my people*.*—*Facilitator

The type of activities also supported satisfaction with the technology club. Facilitators spoke of adolescents enjoying the technology club because they “*excel at this activity*.” Adolescents spoke about how they just “*enjoy learning about coding*” and how it “*feels great to be a coder*.” Parents described their children as motivated to attend the club, with many reporting it was “*the first thing I’ve been able to get them to out of the house… or leave them alone or bring them willingly*.” Parents reported that other extracurricular programs did not suit their child and that they had difficulty finding something their child found interesting.

*I think it’s just because it [technology club] just suits him*. *He’s not really a sporty kid*. *So*, *doing weekend winter sports and summer sports*, *and all that stuff*. *This has been good for him because he’s enjoyed the company of other kids*, *and just being able to get out and have time away from his sisters*. *It’s something for him*, *himself*. *So*, *it’s been really good in that aspect*.*—*Parent

Overall, participants were satisfied with the club because they felt it provided a safe environment where adolescents could access and enjoy technology-related activities.

#### Demand

The demand for the technology club was supported by focus group findings. Thematic analysis revealed one theme related to demand: resources.

The theme of resources relates to demand within the program, adolescents expressed wish for more resources, including activities, hardware, software, and facilitators. Adolescents spoke about wanting to have more “*robot things*” available, “*more helpers*” available, “*more games to play*,” “*more [instruction] books*,” “*MacBook’s for programming*,” and that sessions should last “*three hours not two hours*.” Parents spoke of having longer coding sessions, additional programs, and increasing the number of facilitators. Parents also expressed that it would have been useful to have more parent mentors, saying that it “*would be good to let the parents know about mentorship*, *especially parents on the spectrum themselves*,” with current autistic facilitators described as “*great mentors*.” Parents reinforced that their child enjoyed the program and wished the sessions were longer:

*[My daughter] loves everything about it*. *The only thing she says is its too short*.*—*Parent

Participants’ eagerness to have access to more resources, longer sessions, and additional holiday programs demonstrates the demand for this program.

#### Practicality

Practicality was explored through focus groups with facilitators providing feedback on aspects of implementation. Thematic analysis revealed two themes related to practicality: technology management and facilitator technical knowledge.

The theme, *technology management*, related to the practical issues associated with managing the laptops and software required for the club. The technology club was held in a standard classroom, requiring the setup and pack-up of laptops before and after every session. The reliance on individual laptops for students resulted in practical issues regarding maintaining and managing software, with facilitators expressing that it would have been “*far easier to manage [the computers] centrally*, *a proper computer lab would be awesome*.” A facilitator expressed their frustration at the current system:

*At the moment*, *how we manage the system here is all individual*. *Each machine*. *So*, *everything you do*, *you’ve got to go around and do to each machine… the first half of the term was me running around trying to get something working*.*—*Facilitator

*Practicality* was also impacted by the facilitators’ technical knowledge of software programs used within the club, with facilitators noting that when they were unable to answer a technical question, adolescents became “*frustrated*.” Facilitators took the view that “*with IT (information technology)*, *it’s so big you can never know everything*,” noting that the autistic adolescents always “*push[ed] far beyond any sort of training*.” In working with the adolescents, facilitators modelled collaborative strategies, suggesting “*figure[ing] this out together*” that not having all the answers was “*part of that journey to coding*.” Despite facilitators expressing that at times they felt like they needed more technical knowledge, adolescents described the facilitators as “*very helpful*,” “*friendly*,” and as “*always giving you the help that you want*.” The club provided a safe learning environment to the adolescents, where “*there’s no right or wrong*,” and facilitators would not “*get all angry at you and tell you what to do*.” Regardless, some facilitators felt they lacked technical knowledge, and the practicability of the technology club could be improved by reviewing the facilitator training procedures:

*You can feel it*. *It’s the kid sitting there with that frustration*, *that you’re the mentor*, *you should know what this thing is doing*, *‘You’re the adult*, *you’re supposed to be giving me direction*!*’ This is whether this kid is autistic or not*, *there’s an expectation that you as the adult actually know how this works*.*—*Facilitator

The practicality of the club could be improved by improving the efficiency of managing the hardware and software. The mixed opinions of facilitators surrounding technical experience warrant a review of technology training procedures.

#### Adaptation

Thematic analysis revealed two themes related to adapting the technology club: understanding the individual and visual resources. The theme of *understanding the individual* described tailoring classes to the unique learning needs of each adolescent. Parents commented that if their child got “*stuck or something*,” they would not persist with the activity. Adolescents reinforced that if they ‘failed’ at something, they would not try similar activities because they “*knew they would fail at it again*.” Facilitators highlighted that the club could be “quite chaotic” with “kids at different levels” at times. Facilitators suggested that a complete understanding of each adolescent’s technology experience, interests, and sensory needs would enable them to “*start a conversation*” and connect with adolescents. While this information was available, it was not readily accessible, with facilitators suggesting that a quick reference “*profile*” of each adolescent outlining “*whether this kid is anxious*,” their “*triggers*” and “*interests*” would have been helpful. In addition, facilitators suggested dividing the classroom based on the adolescent’s technology skill level.

*You had kids who were a little bit more advanced*, *whereas other kids were brand new to it*. *I thought it maybe would have been better if the groups were split into medium*, *advanced*, *something like that*.*—*Facilitator

*Adapting* visual resources could also improve the feasibility. Adolescents’ preference for visual resources varied. Each adolescent was provided with a printed manual detailing activities with step-by-step pictures, and while some adolescents found the manuals helpful, others stated they preferred video demonstrations.

*I think that both work good*, *but I would prefer video because then you can just see what’s happening as it’s going*. *Sometimes the books give out where there’s an option or how to activate it*. *But with videos it shows you where it is*.*—*Adolescent

The technology club can be better adapted by providing more readily accessible information to facilitators about adolescent’s individual learning needs and expanding visual resources for teaching.

#### Integration

Thematic analysis revealed two themes relating to how the technology club could be integrated into the Australian disability system: fees and National Disability Insurance Scheme (NDIS) eligibility. While in the current study, autistic adolescents were able to attend the technology club without charge due to funding obtained from an industry sponsor. In discussing the club’s long-term sustainability, parents discussed charging fees to support the maintenance of hardware and software. Parents expressed that they would be “*happy to put my hand in my pocket*” and pay a small fee to attend, comparing the club to other extracurricular activities: “*anything else*” like “*tennis is 12 bucks a week*”. Parents also stated that adding a fee might change how the adolescents viewed the club.

*If the kids are contributing a little bit each week*, *it creates a bit of respect there too*. *Because it is free it doesn’t mean it’s not important*. *It doesn’t mean that it is not valuable*.*—*Parent

*Integration* of the club was impacted by the broader context of the therapy autistic adolescents received. In the Australian context, funding for purchasing disability services and supports provided through the NDIS. While parents mentioned that they would be willing to use their NDIS funding to contribute to the technology club’s costs, under the current rules, the technology club was ineligible as it was “*not run by an approved provider*,” but volunteers. Even though the technology club was not eligible to access NDIS funds, one parent described how it influenced her son’s NDIS plan.

*I just decided that he*, *on his NDIS plan*, *we have just had his new plans drawn up and then he’s decided that he doesn’t need a support worker anymore*. *He doesn’t want to go out with a support worker on a Saturday … he wants to come to the technology club*.*—*Parent

The results indicated that the technology club could be integrated into the current health system by charging a fee to families or becoming an approved NDIS provider in Australia.

#### Expansion

Thematic analysis revealed one theme for expansion: including neurotypical peers When speaking about expanding the club, parents focused on whether the club should be opened up to neurotypical peers or remain exclusive for autistic adolescents. ‘Opening up’ the technology club was seen by parents as potentially providing an opportunity to educate the community on neurodiversity and encourage their autistic children to “*learn how to adapt*,” develop “*resilience*,” and “*give [their autistic children] an insight into life after school*.” However, some parents strongly advocated that the club should remain exclusive to autistic individuals, “*much prefer[ing] it to be a group with just autism*,” allowing their children to have their “*little quirks*,” and be in a space where “*they’re all the same*” and “*they’re not really made fun of*.” These parents worried that opening up the club to neurotypical adolescents would “*open it to bullying*” like at “*mainstream*” school, highlighting that they “*had no bullying here*” and that their child “*loves it for that reason*.” Other parents suggested commencing the club with autistic adolescents only and then opening the club to neurotypical adolescents.

*I think being a parent with a child*, *especially being a parent with a neuro-typical child*, *you can coddle those autistic children too much*. *We need to let them learn the world is not going to change for them*. *They need to learn to be a part of that world… That’s what I’m saying*. *What I’m saying is you’re going to have to bring other kids in eventually*.*—*Parent

Like parents, adolescents had a mixed views regarding opening up the club to neurotypical peers. While one adolescent expressed, “*I’m fine with it*,” another felt it “*depends on the people … [only if they are] friendly and safe*.” Another adolescent strongly felt that the technology club should “*not be open to everybody … I really like being at a coding club with people with autism like me*.” Another adolescent was specific in their recommendation:

*I say we could open it up to other kids that have disabilities too*, *but not to get everyone*. *Because we all share something with our disability*. *Like*, *we’re all different to other people*, *so I think we should all share that*.*—*Adolescent

Expansion of the technology club to include individuals without autism requires careful consideration given parents’ concern of potential bullying.

### Preliminary feasibility

The feasibility of the strength-based technology club was tested through both qualitative and quantitative methods. Thematic analysis of the focus groups revealed three potential outcomes for adolescents: making friends, socializing, and developing positive traits. Quantitative data also informed understanding the feasibility of this approach, even though feasibility studies often do not have large enough sample sizes to produce significant results [64]. While statistical significance may not be achieved, valuable information can be gained about the utility of the measurement framework [65].

#### Qualitative findings

Adolescents, parents, and facilitators highlighted the importance of the technology club in providing opportunities for adolescents to make friends, with parents witnessing the development of “*a lot of friendships*” that “*led to play dates*.” Adolescents spoke of not knowing anyone when they started but eventually “*made friends with a lot of other people*.” While adolescents would normally “*hang out with their parents*,” feeling “*very shy*,” they felt like the club was “*really different*,” and they “*liked hanging out with everyone*.” Parents described the technology as providing a place where “*like-minded*” individuals can “*form friendships outside of school*.” One parent described how because of the club; their child had had their first sleepover at a friend’s place.

*They came here and they’ve just hit it off and so much as to say Chris been at our house for a night*, *Jake’s been to his house for the night*. *They’re 13 years-old*, *that’s Jake’s first ever sleepover*. *So*, *it proves to me that this is a good project*.*—*Parent

Parents and facilitators reported that the technology clubs provided opportunities for adolescents to socialize. Parents said adolescents were “*drawn to the topic not the person*” and if “*they are interested in the topic*” that “*gives them something to talk about*.” Facilitators reported that adolescents were not “*forced [into] social engagement*” and that adolescent’s similar interests acted as a “*natural steppingstone*” to socializing.

*The things that these children have in common*, *they have in common because they excel at this activity*. *That’s why they’re all here and why they’re all enjoying it and why they’re starting to socialise with each other*.*—*Facilitator

Facilitators reported outcomes relating to developing positive traits such as “*bigger picture*” outcomes, including “*learning coping strategies*,” “*confidence*,” and “*problem-solving*.” Adolescents reported similar outcomes, with one adolescent saying, “*I’m confident in doing everything now*.” Parents also noted that the club provided opportunities for adolescents to “*learn how to find those solutions for themselves*.” Parents described their adolescent’s improvements in self-confidence, with advances in “*start[ing] a conversation with people that we haven’t been in contact with*” and confidence when talking with others.

*I think it’s been great that you’ve all brought that up*, *because to me*, *definitely I see it as*, *you’re getting the kids*… *this has been my teaching experience*, *the work is the coding*, *but the bigger picture is that they’re learning coping strategies*, *socialization*, *they’re not alone and that to me is where I’ve really noticed that they’ve grown and flourished*. *It’s a really good programme for the bigger picture stuff*, *which I’m sure they’ll appreciate and reflect on one day*, *but for now they like coding*.—Facilitator

#### Quantitative findings—Adolescent reported

A Wilcoxon signed-rank test showed that post-test domain-specific self-efficacy scores were statistically significantly higher than pre-test domain-specific self-efficacy scores for the subscales of technical knowledge (*z* = -2.667, *p* = 0.008) and explain to others (*z* = -2.046, *p* = 0.041) (Table 6). The technical knowledge subscale included questions such as, “*I feel confident that I can write working code in the computer language of my choice*.” The ‘explain to others’ subscale included questions such as, “*I feel confident that I can explain to a mentor how I completed my coding activity*.” All other CoderDojo self-efficacy subscale scores had higher post-test means; however, the change was not significant.

**Table 6:** Wilcoxon signed-rank test results of adolescent reported measures.

Measure	Mean (SD)		z	p	ES
	Pre-test	Post-test			
Domain-specific self-efficacy^a^					
Total	1445.55 (198.61)	1788.45 (696.55)	-1.6	0.11	
Working Independently	319.55 (78.55)	381.36 (124.69)	-1.78	0.074	
Solving with help	361.82 (49.81)	397.82 (149.84)	-0.71	0.477	
Asking for help	223.36 (76.47)	259.82 (97.69)	-1.11	0.266	
Self-regulation (environment)	296 (77.35)	336.64 (180.91)	-1.02	0.306	
Technical knowledge	139.73 (128.69)	242.36 (165.01)	-2.67	0.008*	-0.569
Explain to others	105.09 (78.01)	170.45 (109.87)	-2.05	0.041*	-0.436
CSIE^a^					
Assert (PA)	4.57 (2.40)	5.39 (2.56)	-1.66	0.097	
Assert + Connect (NO)	4.6 (2.11)	5.64 (2.38)	-2.15	0.032*	-0.457
Connect (LM)	5.18 (1.90)	5.91 (2.84)	-0.89	0.374	
Connect + Yield (JK)	6.86 (1.78)	6.36 (2.95)	-0.04	0.965	
Yield (HI)	5.45 (1.42)	5.14 (2.73)	-0.58	0.563	
Yield + Distance (FG)	5.34 (2.36)	5.05 (2.38)	-0.51	0.609	
Distance (DE)	4.30 (1.50)	4.61 (2.16)	-0.98	0.327	
Distance + Assert (BC)	4.41 (2.50)	4.68 (2.16)	-0.31	0.759	
GSE^a^	24 (4.20)	26.09 (6.30)	-1.34	0.18	
AIR Self-determination^a^					
Total	61.64 (15.53)	63.36 (16.73)	-0.4	0.689	
Things I do	18.45 (5.82)	19 (6.74)	-0.28	0.778	
How I feel	19.91 (5.77)	20.64 (6.10)	-0.21	0.838	
What happens at home	23.27 (5.57)	23.73 (4.82)	-0.28	0.779	
Think	21.63 (4.52)	22.18 (5.40)	-0.12	0.905	
Do	19.45 (6.42)	20.36 (5.97)	-0.71	0.476	
Adjust	20.55 (4.95)	20.82 (5.51)	-0.14	0.889	
PedsQL^TM^ ^a^					
Physical	71.31 (20.91)	70.74 (18.45)	-0.56	0.574	
Emotion	57.73 (24.02)	48.18 (21.36)	-1.32	0.189	
Social	56.36 (20.38)	55.91 (23.00)	-0.17	0.865	
School	64.09 (14.29)	60 (18.17)	-0.67	0.506	
Psychosocial Total	59.39 (16.22)	54.70 (18.07)	-0.85	0.398	
STEM career interest^a^					
Science	36.18 (4.58)	35.64 (6.28)	-0.41	0.682	
Technology	42.27 (5.22)	44.64 (7.27)	-1.31	0.19	
Engineering	34.64 (5.37)	33.36 (12.29)	-0.12	0.906	
Mathematics	39.36 (5.97)	40.55 (6.20)	-0.31	0.758	
Perth Friendship Scale					
Friendship^a^	24.73 (7.54)	23.82 (8.65)	-0.18	0.858	
Solitude negative^b^	19.73 (9.03)	22.73 (10.27)	-1.02	0.306	
Solitude positive^a^	19.73 (6.28)	19.91 (6.61)	-0.31	0.755	
Isolation^b^	13.45 (7.49)	14.91 (7.13)	-1.20	0.23	
Friendship quality scale					
Companionship^a^	11.11 (3.66)	11.78 (3.07)	-0.21	0.833	
Conflict^b^	7.78 (2.95)	9.33 (2.78)	-1.41	0.159	
Help^a^	20.33 (6.58)	18.67 (4)	-0.49	0.622	
Security^a^	17.78 (4.27)	19.22 (6.26)	-0.70	0.483	
Closeness^a^	23.33 (5.48)	23.33 (6.25)	-0.07	0.944	
UCLA Loneliness Scale (ULS-8)^a^					
Total	16.64 (4.13)	17.18 (4.96)	-0.53	0.595	

CSIE: Circumplex Scales of Interpersonal Efficacy; GSE: General Self-efficacy Scale; AIR: American Institutes for Research Self-determination scale; PedsQL^TM^: Pediatric Quality of Life Inventory Version 4.0; STEM: Science, Technology, Engineering and Mathematics; UCLA: University of California, Los Angeles; ES: Effect Size

*p < 0.05

^a^Increasing scores = improvement

^b^ Decreasing scores = improvement

A Wilcoxon signed-rank test showed that post-test CSIE scores were statistically significantly higher than pre-test CSIE scores for the subscale of Assert and Connect (NO) (*z* = -2.145, *p* = 0.032) (Table 6). The Assert and Connect subscale included questions such as, “*when I am with others*, *I can express myself*,” and “*when I am with others*, *I can be a leader*.” There was no further statistically significant change in adolescent self-reported measures at post-test.

#### Quantitative findings–Parent reported

A Wilcoxon signed-rank test showed that post-test AIR self-determination scores were statistically significantly higher than pre-test AIR self-determination scores for the sub scales of DO (*z* = -2.001, *p* = 0.045) (Table 7). The DO subscale relates to making choices and plans to meet goals and taking action to complete plans (AIR Self-determination manual); for example, “*my child figures out how to meet goals alone*. *(S)he makes plans and decides what to do independently*”. While the mean of all other AIR self-determination scale scores were higher at post-test, there was no significant change from the pre-test.

**Table 7:** Wilcoxon signed-rank test results of parent reported measures.

Measure	Mean (SD)		z	p	ES
	Pre-test	Post-test			
PedsQL^TM^ ^a^					
Physical	71.09 (18.50)	70.83 (22.27)	-0.09	0.929	
Emotional	62.5 (22.00)	56.25 (20.46)	-1.38	0.168	
Social	53.33 (28.15)	49.17 (25.30)	-0.07	0.944	
School	58.33 (11.74)	59.17 (16.90)	-0.12	0.905	
Psychosocial total	58.06 (16.99)	54.86 (16.26)	-0.63	0.529	
AIR Self-determination^a^					
Total	38.42 (7.91)	42.33 (6.43)	-1.89	0.059	
Things my child does	14.5 (5.20)	15.42 (4.69)	-1.66	0.096	
What happens at home	23.92 (3.75)	25.58 (2.75)	-1.75	0.081	
Think	14.58 (2.75)	15.42 (1.78)	-1.14	0.254	
Do	11.75 (3.25)	13.5 (3)	-2.00	0.045*	-0.408
Adjust	12.083 (2.43)	13.42 (2.19)	-1.85	0.064	

PedsQL^TM^: Pediatric Quality of Life Inventory Version 4.0; AIR: American Institutes for Research Self-determination scale; ES: Effect Size

*p < 0.05

^a^Increasing scores = improvement

^b^Decreasing scores = improvement

A Wilcoxon signed-rank test showed no significant change in post-test PEDSQL scores. All subscales, except school, had lowered post-test scores (Table 7).

### Measurement framework utility

In addition to reporting preliminary efficacy, feasibility studies are useful in determining the measurement framework’s appropriateness, including testing the data collection procedures, the likelihood of participants completing all questionnaires, and the time needed to complete the measurement framework [65]. In line with leveraging interests, the measures were converted to an electronic format that allowed adolescents and their parents to complete the measures online, via a smartphone or computer. There were no noted concerns with the data collection procedures. All measures were considered suitable, given ten out of 11 adolescents completed 100% of the measures. The time to complete measures was recorded automatically using the online program, with an average completion time of 46.2 minutes (*SD* = 29.457) for pre-test and 32.1 minutes (*SD* = 11.120) for post-test for adolescents, and 21.4 minutes (*SD* = 8.488) for pre-test and 18 minutes (*SD* = 6.021) for post-test for parents, excluding one adolescent and three parent outliers.

## Discussion

This study aimed to systematically evaluate a strength-based technology club for autistic adolescents guided by the feasibility focus areas of Bowen et al. [43]. The overall findings revealed that autistic adolescents, their parents, and facilitators were highly satisfied with the technology program *(acceptability)* fundamentally because it fostered a safe environment and provided an opportunity for adolescents to engage in enjoyable activities. There was an obvious *demand* for the technology club, with adolescents expressing a wish for more resources, highlighting that the club provided an opportunity to participate in activities not available elsewhere. The potential for *integrating* the club into the current health system was demonstrated, as parents expressed a willingness to pay a fee or access NDIS funding if the technology club was an approved service provider. The feasibility areas that required further review include *practicality*, *adaptation*, *expansion*, and *preliminary efficacy*.

*Acceptability* and *Practicality* of the technology club could be improved through better technology management, specifically overcoming the challenges associated with using individual laptops. The technology club relied on industry funding to provide the laptops and other equipment for the club. The laptops provided the opportunity to install new software programs without network restrictions, allowing facilitators to align software with individual autistic adolescents’ interests, strengths, and goals, enabling autonomy and applying an individualized approach. While the laptops afforded greater flexibility in relation to software, the facilitators provided feedback that maintaining and setting up the laptops was very time intensive. Facilitators recommended that the laptops be networked, allowing them to be managed centrally. It is suggested that future technology clubs secure a venue that provides a networked computer laboratory. Investigation of technology clubs [42] held in computer laboratories within tertiary institutions highlighted that while computer laboratories may have restrictions with installing new software, the computers are more readily managed.

The *practicality* of the club was impacted by the facilitator’s technical knowledge, and while some facilitators expressed a desire for more training regarding the software enabling them to support the adolescents, others felt that you could never know everything when it comes to technology. Future programs could be improved by providing more facilitator training on software, collaborative teaching practices, and strategies focusing on promoting autonomy in autistic adolescents. The technology club evaluated in the present study provided adolescents with the choice of four activities: making a computer game, coding a text adventure, online coding activities, and robotics. While the intention of providing these choices was to foster autonomy, it presented challenges for facilitators, requiring them to have proficiency across multiple programs. Previous strength-based technology programs described in the literature commonly provide the opportunity for participants to learn one software program with instruction provided by an expert [34, 40, 63–67]. In these studies, autonomy was fostered by allowing students to make choices within the activity. For example, in a program teaching 3D modelling, adolescents created a design of their choice, such as a dinosaur, a house, or a train. Previous research suggests that opportunities for choosing activities reduce autistic adolescents challenging behaviours and promotes their engagement [68–72], so this study provided across-activity choice, meaning adolescents could choose from multiple activities. While it is recommended that adolescents be allowed to choose both across and within activities, delivering these opportunities requires a significant investment in training facilitators. Facilitators should also adopt a youth-centred learning approach, learning with adolescents, respecting their needs and abilities, providing an individualized approach, and following their lead [40]. In line with previous research, the present study highlighted the importance of facilitators building rapport with adolescents to reduce problem behaviours and increase social interaction [73]. Autistic adolescents value the facilitators of strength-based technology clubs not only for their technical skills but for being approachable and presenting as role models rather than authority figures [35]. Treating adolescents as equals is a common theme within the strength-based technology literature [34, 35]. In retrospect, the program’s facilitators evaluated in the present study would have benefited from further training, particularly given the range of activities delivered. Our findings also point to the importance of ensuring facilitators have the appropriate technical skills, are also approachable and friendly, and adopt an egalitarian teaching style.

Future iterations of the club could be improved by reviewing the *adaptation* themes. Facilitators provided feedback that they did not readily have access to pre-assessment information pertaining to adolescents’ interests, strengths, previous technology experience, sensory needs, and learning preferences, limiting their understanding of individual student needs. Smaller working groups and creating participant ‘profile cards’ detailing autistic adolescents’ sensory preferences, special interests, and abilities, were suggested by facilitators as strategies to improve individualized teaching. Adapting teaching to sensory preferences, special interests, and abilities is well supported in the literature [50, 74–76]. Smaller working groups also provide opportunities for building rapport, one-on-one learning, increasing adolescents’ activity engagement [77], and opportunities for peer learning [78]. While increasing the number of individual teaching strategies is recommended, technology clubs should continue to facilitate a flexible low-pressure learning environment [35].

The feasibility of the club could be improved by *adapting* the visual resources provided. While each adolescent was provided with a coding manual with visual illustrations that aimed to leverage the visual processing strengths of autistic individuals [76], adolescents’ expressed that they would appreciate video instructions detailing each step. Future programs could utilize materials readily available through sources such as YouTube to support problem solving and activity engagement.

In regard to *demand and expansion*, Parents and adolescents presented mixed views with *expanding* the club, particularly concerning whether neurotypical adolescents should attend. While acknowledging the benefits of inviting neurotypical adolescents into the club, parents also recognized that their children enjoyed the club because it provided a safe space where everyone had an autism diagnosis [33]. Sharing the experience of an autism diagnosis with other adolescents fosters a sense of belonging [33, 42]. Given that for autistic adolescents, shared interests are a powerful motivator of participation, socializing, and belonging [33, 35, 40], inviting other adolescents into a technology club who share the same interest could foster feelings of belongingness regardless of their diagnostic status.

Another potential option to *expand* the club to neurotypical adolescents while maintaining a feeling of safety and belonging would be to increase family collaboration. Inviting autistic adolescents’ siblings into technology clubs has been noted as an effective strategy in promoting positive outcomes [33, 79], including fostering feelings of pride in their autistic siblings’ strengths and abilities [33, 66], and improving communication and interaction between siblings [33, 66]. Service providers should consider starting technology clubs exclusive to autistic adolescents to facilitate belonging and a safe environment but later expand the club to include neurotypical adolescents through family collaboration and those who share a genuine interest in technology activities.

### Preliminary efficacy

Preliminary efficacy was assessed via qualitative and quantitative methods. The focus groups identified that the technology clubs fostered outcomes, including making friends, opportunities to socialize, and developing positive traits. Preliminary efficacy testing highlighted adolescents increased self-efficacy in their technology skills and interpersonal skills, specifically in asserting themselves while being cooperative. While preliminary, these findings were triangulated with qualitative data that reported adolescents increased social interaction.

Expected improvements in self-determination, quality of life, and friendship were not demonstrated and were likely impacted by the small sample size. While statistical significance was not demonstrated on a number of measures, this is not unexpected for a feasibility study [80]. Non-significant results with feasibility studies are not an indication of poor feasibility [80]. The purpose of preliminary efficacy testing is to design a more rigorous evaluation [65] and assess the utility of the measurement framework [43]. The present study demonstrated the utility of the measurement framework given the high completion rate and low time to complete, suggesting it would be appropriate for more rigorous efficacy studies.

## Limitations

While the present study’s findings are promising, they should be considered in the context of several limitations. Firstly, the nature of the club and its focus on computer coding and technology meant that club recruitment was directed at towards autistic adolescents without any confirmed intellectual disability because of the available activities, resourcing, and the criteria of the club. Secondly, adolescent’s autism diagnosis was based upon parent report and the SRS-2 [50]. Ideally, diagnoses should be verified via medical report, with additional measures employed, such as the Autism Diagnostic Observation Scale (ADOS) [81], which has high sensitivity and specificity [82]. Qualitative data was limited to autistic adolescents who continued with the program and did not capture the experiences of adolescents who left the technology club following the holiday program. Limitations also exist within the assessment framework. The P values were not corrected for multiple comparisons as this was a feasibility study using both qualitative and quantitative method to provide an initial basis of future research. We provided limited efficacy testing and evaluation of measurement framework. Not all measures had demonstrated psychometric properties for autistic adolescents and the time taken to complete the measures may have been a deterrent to autistic adolescents. Future research should carefully consider balancing measuring outcomes and the burden it places on participants.

## Conclusion

This feasibility study provides valuable information relating to the feasibility of a 15-week strength-based technology club for autistic adolescents, providing a basis for future efficacy studies. The autism literature is dominated by deficit-based models and this research contributes to the limited studies supporting a strength-based approach. While this study specifically employed technology activities, not all outcomes and strategies are specific to technology use. For example, creating a safe environment and leveraging adolescents’ interests are applicable to all intervention programs. Further testing of these strategies with larger sample sizes will justify their importance in strength-based programs and encourage the technology industry to focus on strengths rather than deficits when aiming to improve the outcomes for autistic individuals.

## Supporting information

S1 File Example interview discussion guide questions. (DOCX)

S2 File Example of visual prompts used with participants. (DOCX)

S3 File List of quantitative measures used in study. (DOCX)
