# Supplementary material for: Strength-based technology clubs for autistic adolescents: A feasibility study
Source: PLoS One. 2023 Feb 3;18(2):e0278104. doi: 10.1371/journal.pone.0278104 (PMC9897531; doi:10.1371/journal.pone.0278104)
Supplement: S1 File — (DOCX) [file pone.0278104.s001.docx]

**SUPPLEMENTARY INFORMATION S1**

**S1.** **Example interview discussion guide questions**

|  | Example questions | | |
| --- | --- | --- | --- |
| Feasibility topic | Adolescent | Parent | Facilitators |
| Acceptability and demand | What did you enjoy most?  What did you NOT enjoy?  What was the hardest thing about the computer coding club? Why/can you give an example? | What do you believe your child enjoyed most? Why?  What do you think your child enjoyed least? Why?  What has your experience been with the club? | What was your experience like teaching adolescents with autism?  What was the hardest part about teaching at the coding club? |
| Practicality and adaptation | If you were in charge of the computer coding club, what is one thing you would change? Why?  What activities would you like to do more of? Why?  Are there any technology activities that you would like to try in the future? | Which coding activity did your child enjoy the most? (Construct 2, Twine, Robotics, Online coding) Why?  What did you think about group lunch time?  Did the activities appeal to your child’s interests? How? | Would you add any more coding activities? If yes, what and why?  Would you remove any coding activities? If yes, what and why? |
| Implementation and Adaptation | Did you feel the facilitators knew a lot about technology and coding?  What could we do more of, to help you learn?  How did you like the coding manuals provided? | What is the most important information facilitators should know about your child before they attend the club? Why?  What made learning easier for your child?  What made learning harder for your child? | Do you feel you received adequate training before starting the coding club? If no, what other training is required?  What strategies helped the students learn?  What strategies could be improved to help learning? |
| Perceived outcomes | Did you have any coding goals? If so, did you achieve them?  What did you learn from the computer coding club?  Have you made any friends at the club? If yes, provide example of how you made a friend. | What was the biggest impact the coding club had on your child?  What was the biggest impact the coding club had on your family?  What technology or coding skills did your child learn? | What are the benefits of the coding club?  Are there any negative outcomes from attending the club?  What technology or coding skills did the students learn? |
| Integration and expansion | Would you be happy for kids without autism to join the club? | Has the club impacted your use of autism funding? | What do you think about the club only being open to adolescents with autism? |
