# Supplementary material for: Strength-based technology clubs for autistic adolescents: A feasibility study
Source: PLoS One. 2023 Feb 3;18(2):e0278104. doi: 10.1371/journal.pone.0278104 (PMC9897531; doi:10.1371/journal.pone.0278104)
Supplement: S2 File — (DOCX) [file pone.0278104.s002.docx]

**SUPPLEMENTARY INFORMATION S2**

**S2. Example of visual prompts used with participants**

Experience

*
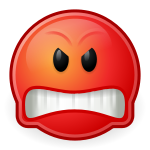

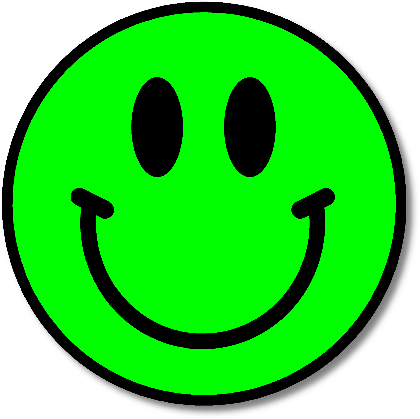
*

General feedback

What did you enjoy most?

What did you NOT enjoy?

Activities

*
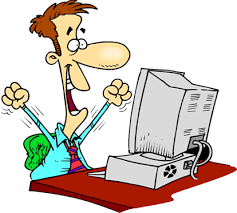
*

What activities would you add?

What activities would you remove?

What did you like about the holiday program?

What did you like about Construct 2 (video game making), Twine (multi choice ending) CodeCombat (online coding) and the robots?

Did you enjoy presenting your work? Why?

Mentors


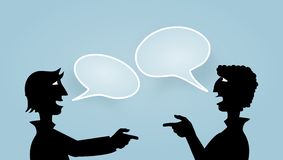


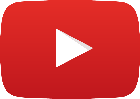


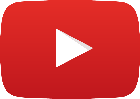


Did you feel comfortable speaking with the facilitators? Why or why not/examples?

Did you feel the facilitators knew a lot about technology and coding?

What made learning easy?

What made learning hard?

How did you like the coding manuals provided?


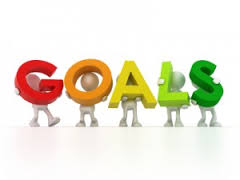
Outcomes

What did you learn from the computer coding club?

Did you have any coding goals? If so, did you achieve them?

Outcomes – confidence, belonging, motivation, developing technology skills, frustrated, proud, friendship.
