# Supplementary material for: Strength-based technology clubs for autistic adolescents: A feasibility study
Source: PLoS One. 2023 Feb 3;18(2):e0278104. doi: 10.1371/journal.pone.0278104 (PMC9897531; doi:10.1371/journal.pone.0278104)
Supplement: S3 File — (DOCX) [file pone.0278104.s003.docx]

**SUPPLEMENTARY INFORMATION S3**

**S3.** **List of quantitative measures used in study**

Domain-specific self-efficacy scale

Informed by Bandura’s [83] guide for constructing self-efficacy scales, a domain-specific self-efficacy scale was developed. Adolescents completed a 32-item questionnaire examining the confidence in completing certain technology activities, with values ranging from zero (not confident at all) to 100 (absolutely confident). The 32-items contributed to six subscales informed by several published technology and computer self-efficacy scales [84–87] covering the areas of working independently, solving with help, asking for help, self-regulation, technical knowledge, and explaining technology to others. Higher scores indicate higher self-efficacy for the technology club activities.

**Circumplex Scales of Interpersonal Efficacy (CSIE)**

Adolescent’s confidence in successfully performing interpersonal behaviours was assessed via the Circumplex Scales of Interpersonal Efficacy (CSIE) [88]. Adolescents indicated their confidence in interpersonal behaviours by completing 32 questions using an 11-point scale, with values ranging from zero (I am not at all confident that…) to 10 (I am absolutely confident that...). Higher scores indicate greater interpersonal efficacy, divided into eight sectors; assert, assert and connect, connect, connect and yield, yield, yield and distance, distance and distance and assert. The measure has demonstrated good psychometric properties and has been used by the authors with autistic adolescents [89].

**General Self-efficacy Scale (GSE)**

The General Self-efficacy Scale (GSE) [90] was used to measure global self-efficacy through adolescent self-report. Adolescents responded to 10-items using a four-point Likert scale with values ranging from one (Not at all true) to four (Exactly true). The GSE Scale items are unidimensional [90] and previously used to investigate employment outcomes for autistic adults [91,92].

**American Institutes for Research Self-determination scale (AIR)**

Adolescents' capacity and opportunity to be self-determined were assessed using the AIR self-determination scale [93] via adolescent self-report and parent proxy report. Adolescents responded to 18 questions relating to decision making (e.g., I know what I need, what I like, and what I’m good at) on a five-point Likert scale with values ranging from one (Never) to five (Always). Demonstrating good reliability (alternative item, split-half, and test-retest) and validity (construct validity) in students with and without disabilities (n = 450, 6–25 years old) [64], the AIR has also demonstrated sensitivity to change in students with a disability [94]. The AIR has been shown to be reliable and valid for measuring self-determination in autistic populations [95]. The section assessing opportunities for self-determination at school was removed given the technology club did not aim to impact the school environment, and parent report of self-determination opportunities at school are unreliable [96].

Paediatric Quality of Life Inventory Version 4.0 (PedsQLTM 4.0)

Quality of life (QoL) was assessed by parent proxy and adolescent self-report using the 23-item Paediatric Quality of Life Inventory Version 4.0 (PedsQLTM 4.0), covering four QoL domains: physical, emotional, social, and school. A five-point Likert scale was employed with values ranging from zero (never a problem) to four representing (almost always a problem). Lower scores indicate better quality of life. The PedsOLTM is the most frequently used quality of life measure for autistic children [97], with previous studies demonstrating high internal consistency in autistic adolescents (Cronbach’s alpha = 0.89) [98].

**Science, Technology, Engineering and Mathematics Career Interest Survey (STEM-CIS)**

Adolescents reported career interest in science, technology, engineering, or mathematics fields was assessed via the STEM Career Interest Survey (STEM-CIS) [99]. Adolescents completed 44 questions relating to self-efficacy, goals, interests, supports, and barriers within each career field (e.g., I plan to use science in my future career). A five-point Likert scale was utilized with values ranging from one (Strongly Disagree) to five (Strongly Agree), with higher scores indicating more interest in a career in science, technology, engineering, or mathematics. The four subscales of science, technology, engineering, and mathematics can be administered separately or in combination, representing strong construct validity [99].

**Perth A-Loneliness Scale (PALS)**

The Perth A-Loneliness scale (PALs) [100] seeks adolescents’ responses to 24 items across four factors (isolation, friendship, positive attitude to solitude, and negative attitude to solitude) rated on a six-point Likert scale ranging from one (never) to six (always). Higher scores for friendship and positive attitude to solitude indicate less loneliness, with higher scores for isolation and a negative attitude to solitude indicating increased loneliness. The internal consistency and construct validity has been established in a sample of 1143 adolescents without disability [101].

**University of California, Los Angeles (UCLA) Loneliness Scale - short version (ULS-8)**

The University of California, Los Angeles (UCLA) Loneliness Scale [102] is a widely used self-report measure of loneliness, with a shortened version of eight questions available [103], scored on a four-point Likert scale with values ranging from one (never) to four (always) with higher scores indicated greater loneliness. The scale is homogenous in measuring loneliness and demonstrates strong validity and reliability in tertiary education populations [102,103]. The ULS-8 has also been used in multiple studies with autistic samples showing good internal consistency [104].

**Friendship Qualities Scale**

The Friendship Qualities Scale [105] is a self-report 23-item questionnaire measuring perception of friendship quality in relation to a best friend. The 23 items are scored on a six-point Likert scale with values ranging from one (never) to six (always), forming five categories: companionship (voluntary time spent together), conflict (arguments or disagreements with friend), help (helping each other with problems), security (trust in friendship and continuing friendship through conflict), and closeness (appreciation and acceptance by friend) [105]. The Friendship Qualities Scale is commonly used with autistic children [9,10,106,106], and previous studies have demonstrated a high internal consistency [10].

**References**

83. Bandura A. Guide for constructing self-efficacy scales. In: Self-efficacy beliefs of adolescents. Charlotte, NC: Information Age Publishing; 2006. p. 307–37.

84. Askar P, Davenport D. An investigation of factors related to self-efficacy for java programming among engineering students. Turkish Online J Educ Technol. 2009;8(1):26–32.

85. Korkmaz Ö, Altun H. Adapting computer programming self-efficacy scale and engineering students’ self-efficacy perceptions. Particip Educ Res [Internet]. 2014 Jun 1 [cited 2020 Sep 9];1(1):20–31. Available from: http://www.perjournal.com/archieve/issue_1_1/2-per_14-02_volume_1_issue_20_page_1_31.pdf. doi:10.17275/per.14.02.1.1

86. Ramalingam V, Wiedenbeck S. Development and validation of scores on a computer programming self-efficacy scale and group analyses of novice programmer self-efficacy. J Educ Comput Res [Internet]. 1998 Dec [cited 2020 Sep 9];19(4):367–81. Available from: http://journals.sagepub.com/doi/10.2190/C670-Y3C8-LTJ1-CT3P. doi:10.2190/C670-Y3C8-LTJ1-CT3P

87. Yukselturk E, Altiok S. An investigation of the effects of programming with Scratch on the preservice IT teachers’ self-efficacy perceptions and attitudes towards computer programming. Br J Educ Technol [Internet]. 2017 May [cited 2020 Sep 9];48(3):789–801. Available from: http://doi.wiley.com/10.1111/bjet.12453. doi:10.1111/bjet.12453

88. Locke KD, Sadler P. Self-efficacy, values, and complementarity in dyadic interactions: Integrating interpersonal and social-cognitive theory. Personal Soc Psychol Bull [Internet]. 2007 Jan 2 [cited 2020 Jan 31];33(1):94–109. Available from: http://journals.sagepub.com/doi/10.1177/0146167206293375. doi:10.1177/0146167206293375

89. Locke KD, Mitchell GE. Self-perceptions, parent-perceptions, and meta-perceptions of the interpersonal efficacy of adolescents with autism spectrum disorder. Res Autism Spectr Disord [Internet]. 2016 Nov [cited 2020 Jan 19];31:19–29. Available from: https://linkinghub.elsevier.com/retrieve/pii/S1750946716300861. doi:10.1016/j.rasd.2016.07.006

90. Schwarzer R, Jerusalem M. Generalized self-efficacy scale. In: Weinman J, Wright S, Johnston M, editors. Measures in health psychology: A user’ portfolio Causal and control beliefs. Windsor, UK: NFER-NELSON; 1995. p. 35–7.

91. Lorenz T, Heinitz K. Aspergers – Different, not less: Occupational strengths and job interests of individuals with Asperger’s syndrome. Dichter GS, editor. PLoS One [Internet]. 2014 Jun 20 [cited 2021 Mar 24];9(6):e100358. Available from: https://dx.plos.org/10.1371/journal.pone.0100358. doi:10.1371/journal.pone.0100358

92. Lorenz T, Frischling C, Cuadros R, Heinitz K. Autism and overcoming job barriers: Comparing job-related barriers and possible solutions in and outside of autism-specific employment. Hadjikhani N, editor. PLoS One [Internet]. 2016 Jan 14 [cited 2021 Mar 24];11(1):e0147040. Available from: https://dx.plos.org/10.1371/journal.pone.0147040. doi:10.1371/journal.pone.0147040

93. Wolman JM, Campeau PL, DuBois PA, Mithaug DE, Stolarkski VS. AIR Self-Determination Scale and user guide [Internet]. Palo Alto, CA, CA: American Institutes for Research; 1994. Available from: http://www.zarrow.ou.edu

94. Lee Y, Wehmeyer ML, Palmer SB, Williams-Diehm K, Davies DK, Stock SE. Examining individual and instruction-related predictors of the self-determination of students with disabilities: Multiple regression analyses. Remedial Spec Educ [Internet]. 2012 May 20 [cited 2021 Mar 24];33(4):150–61. Available from: http://journals.sagepub.com/doi/10.1177/0741932510392053. doi:10.1177/0741932510392053

95. Chou Y-C, Wehmeyer ML, Skorupski W, Palmer S, Turnbull AP, Smith S. Autism and self-determination: measurement and contrast with other disability groups (Doctoral dissertation). ProQuest Diss Publ. 2013;

96. Carter EW, Lane KL, Cooney M, Weir K, Moss CK, Machalicek W. Self-determination among transition-age youth with autism or intellectual disability: parent perspectives. Res Pract Pers with Sev Disabil. 2013;38(3):129–38.

97 Jonsson U, Alaie I, Löfgren Wilteus A, Zander E, Marschik PB, Coghill D, et al. Annual research review: Quality of life and childhood mental and behavioural disorders - a critical review of the research. J Child Psychol Psychiatry [Internet]. 2017 Apr [cited 2017 Feb 14];58(4):439–69. Available from: http://doi.wiley.com/10.1111/jcpp.12645. doi:10.1111/jcpp.12645

98. McStay RL, Dissanayake C, Scheeren A, Koot HM, Begeer S. Parenting stress and autism: The role of age, autism severity, quality of life and problem behaviour of children and adolescents with autism. Autism [Internet]. 2014 Jul 8 [cited 2020 Sep 11];18(5):502–10. Available from: http://journals.sagepub.com/doi/10.1177/1362361313485163. doi:10.1177/1362361313485163

99. Kier MW, Blanchard MR, Osborne JW, Albert JL. The development of the STEM Career Interest Survey (STEM-CIS). Res Sci Educ [Internet]. 2014 Jun 20 [cited 2020 Mar 8];44(3):461–81. Available from: http://link.springer.com/10.1007/s11165-013-9389-3. doi:10.1007/s11165-013-9389-3

100. Houghton S, Hattie J, Wood L, Carroll A, Martin K, Tan C. Conceptualising Loneliness in Adolescents: Development and Validation of a Self-report Instrument. Child Psychiatry Hum Dev [Internet]. 2014 Oct 15 [cited 2021 Mar 24];45(5):604–16. Available from: http://link.springer.com/10.1007/s10578-013-0429-z. doi:10.1007/s10578-013-0429-z

101. Hays R, DiMatteo MR. A short-form measure of loneliness. J Pers Assess [Internet]. 1987 Mar 1 [cited 2020 Sep 17];51(1):69–81. Available from: http://www.tandfonline.com/doi/abs/10.1207/s15327752jpa5101_6. doi:10.1207/s15327752jpa5101_6

102. Wu C, Yao G. Psychometric analysis of the short-form UCLA Loneliness Scale (ULS-8) in Taiwanese undergraduate students. Pers Individ Dif [Internet]. 2008 Jun [cited 2020 Sep 11];44(8):1762–71. Available from: https://linkinghub.elsevier.com/retrieve/pii/S0191886908000536. doi:10.1016/j.paid.2008.02.003

103. Mazurek MO. Loneliness, friendship, and well-being in adults with autism spectrum disorders. Autism [Internet]. 2014 Apr 3 [cited 2020 Oct 26];18(3):223–32. Available from: http://journals.sagepub.com/doi/10.1177/1362361312474121. doi:10.1177/1362361312474121

104. Bukowski W, Hoza B, Boivin M. Measuring friendship quality during pre- and early adolescence: The development and psychometric properties of the friendship qualities scale. J Soc Pers Relat. 1994;11:471–84.

105. Reichow B, Steiner AM, Volkmar F. Social skills groups for people aged 6 to 21 with autism spectrum disorders (ASD). Cochrane Database Syst Rev [Internet]. 2012 Jul 11 [cited 2020 Aug 10];(7):1–38. Available from: http://doi.wiley.com/10.1002/14651858.CD008511.pub2. doi:10.1002/14651858.CD008511.pub2

106. Calder L, Hill V, Pellicano E. ‘Sometimes I want to play by myself’: Understanding what friendship means to children with autism in mainstream primary schools. Autism [Internet]. 2013 May 27 [cited 2020 Apr 30];17(3):296–316. Available from: http://journals.sagepub.com/doi/10.1177/1362361312467866. doi:10.1177/1362361312467866
